# CAP: The creativity assessment platform for online testing and automated scoring

**DOI:** 10.3758/s13428-025-02761-9

**Published:** 2025-08-18

**Authors:** John D. Patterson, Jimmy Pronchick, Ruchi Panchanadikar, Mark Fuge, Janet G. van Hell, Scarlett R. Miller, Dan R. Johnson, Roger E. Beaty

**Affiliations:** 1 Department of Psychology, The Pennsylvania State University, 140 Moore Building, University Park, PA 16802 USA; 2 School of Computing, Clemson University, Clemson, SC USA; 3 Department of Mechanical and Process Engineering, ETH Zurich, Zürich, Switzerland; 4 Department of Mechanical Engineering, Pennsylvania State University, University Park, PA USA; 5 Department of Cognitive and Behavioral Science, Washington and Lee University, Lexington, VA USA

**Keywords:** Online data collection, Automated creativity scoring, Divergent thinking, Scientific creativity, Visual creativity, Computational psychometrics

## Abstract

Creativity is increasingly recognized as a core competency for the 21st century, making its development a priority in education, research, and industry. To effectively cultivate creativity, researchers and educators need reliable and accessible assessment tools. Recent software developments have significantly enhanced the administration and scoring of creativity measures; however, existing software often requires expertise in experiment design and computer programming, limiting its accessibility to many educators and researchers. In the current work, we introduce CAP—the Creativity Assessment Platform—a free web application for building creativity assessments, collecting data, and automatically scoring responses (cap.ist.psu.edu). CAP allows users to create custom creativity assessments in ten languages using a simple, point-and-click interface, selecting from tasks such as the Short Story Task, Drawing Task, and Scientific Creative Thinking Test. Users can automatically score task responses using machine learning models trained to match human creativity ratings—with multilingual capabilities, including the new Cross-Lingual Alternate Uses Scoring (CLAUS), a large language model achieving strong prediction of human creativity ratings in ten languages. CAP also provides a centralized dashboard to monitor data collection, score assessments, and automatically generate text for a Methods section based on the study’s tasks, metrics, and instructions—with a single click—promoting transparency and reproducibility in creativity assessment. Designed for ease of use, CAP aims to democratize creativity measurement for researchers, educators, and everyone in between.

## Introduction

Creativity is widely acknowledged as an essential 21st-century skill (Cropley & Singh, 2023). This follows from assessments on the future of work, which anticipate that artificial intelligence and automation will play an increasingly larger role in the workforce—automating tasks that are deterministic, clearly defined, and repeatable (e.g., manufacturing, customer service, shipping, telemarketing). Yet, while automation is anticipated to assume an increasing share of the job market, many jobs and skills will remain out of automation’s reach—specifically jobs and tasks that require varied and complex subroutines (e.g., education, business strategy, psychology). Such tasks require innovative solutions that adapt to changing circumstances—in other words, creativity. Accordingly, a growing emphasis of education is to foster creativity and other soft skills that remain difficult to automate (Cropley et al., 2024; Scott-Barrett et al., 2023). Indeed, creativity training programs have been implemented successfully in classrooms around the world (Ritter et al., 2020; Ruiz-del-Pino et al., 2022)

But how can creativity be measured? Researchers have been grappling with this question for the past several decades (for a review, see Said-Metwaly et al., 2017). Many tests of creativity have been developed, ranging from assessments of divergent and convergent thinking (e.g., the Alternate Uses Task [AUT] and the Remote Associates Task; Mednick, 1962) to assessments of figural creativity, to measures of scientific and real-world problem solving (Aschauer et al., 2022; Barbot, 2018; Beaty et al., 2014, 2024; Sak & Ayas, 2013; Urban, 2004). Creative performance is often quantified using approaches involving human evaluation, such as the Consensual Assessment Technique (Amabile, 1982). Here, a cohort of human raters is asked to give subjective creativity judgements for each idea or product; such approaches have been shown to be reliable and valid (e.g., Silvia et al., 2008).

However, creativity assessment is frequently not scalable—particularly for individuals such as educators who have limited time, expertise in psychological assessment, and access to research assistants. Administering creativity tests in a psychometrically valid way requires expert knowledge of the test itself, experimental design, and computer programming software (such as PsychoJS or Qualtrics). Moreover, scoring creativity tasks (and assessments composed of multiple tasks) is often a labor- and time-intensive aspect of measuring creativity, yielding hundreds or thousands of responses that require scoring by multiple raters to achieve high reliability. For those without access to human or financial resources, creativity measurement can be prohibitively expensive.

Fortunately, recent technological advances have facilitated both testing and scoring of creativity assessments. Regarding testing, an array of computer software enables users to build and customize assessments of their choice. These software solutions vary in terms of the technical expertise necessary to construct the study/assessment. Some solutions, like Lab.js (Henninger et al., 2021), PsychoJS/PsychoPy (‘builder view’; Peirce et al., 2022), JsPsych (De Leeuw, 2015), and Qualtrics (Provo, UT) offer relatively approachable graphical user interfaces (GUI) with point-and-click mechanics, while other software solutions, like PsychoJS/PsychoPy, offer greater flexibility, but at the cost of requiring significant programming knowledge. Although these toolboxes are not catered *specifically* to creativity assessment, researchers often use them to administer electronic creativity assessments at scale.

With regard to creativity scoring, recent advances have changed the landscape of how researchers score their data. Specifically, developments in natural language processing and computer vision models enable automated creativity scoring. This scoring revolution has come in two waves. In the first wave, creativity researchers capitalized on unsupervised learning models to approximate creativity scores, such as semantic distance (e.g., SemDis: Beaty & Johnson, 2021; Open Creativity Scoring: Dumas et al., 2021). These unsupervised models are not trained directly to predict creativity scores but, instead, are trained to model distributional properties of the domain they are trained on (e.g., language or images; Bruni et al., 2014; Lenci & others, 2008; Lin et al., 2022). As a result of this unsupervised training, the models learn to represent visual or verbal stimuli in high-dimensional numerical vector spaces, which can then be used to compute semantic distance scores.

Semantic distance (i.e., 1-cosine similarity between word representations) has been used to automatically score verbal creativity tasks, such as the AUT. For example, SemDis computes the distance between a creativity prompt (e.g., the AUT item BRICK) and the provided response (e.g., ‘use it as a doorstop’). Similarly, Divergent Semantic Integration (DSI) computes the pairwise distances between all words in a text, such as short stories (Johnson et al., 2023). Higher semantic distances reflect more remote associations and therefore higher creativity. Indeed, prior work has exhibited that semantic-distance-based approaches provide a good proxy for creativity; semantic distance scores exhibit moderate positive correlations with human creativity ratings at the response level (*r* ~ =.12 to.4; e.g., Organisciak et al., 2023), and even stronger correlations at the participant level (*r* ~ =.45 to.85; Johnson et al., 2023).

More recently, *supervised* scoring methods have led to unprecedented accuracy in matching human creativity ratings (Acar et al., 2024; Cropley & Marrone, 2022; DiStefano et al., 2024; Luchini et al., 2023; Organisciak et al., 2023; Patterson et al., 2024). In supervised learning, models are trained to represent the creativity of responses to a given task by providing responses as inputs and training the model to predict the corresponding human ratings at the model’s output. A subtype of supervised learning, known as transfer learning (Tan et al., 2018), has been key to recent scoring advances. In transfer learning, a ‘pre-trained’ unsupervised model is provided with additional supervised training on a (creativity) task of interest. This allows the model to use its pre-existing distributional knowledge when learning the supervised task, which speeds the rate at which it learns the task and often increases the model’s ultimate performance on the transfer task (e.g., scoring ideas is has not seen before), relative to beginning with a non-pre-trained model (Tan et al., 2018).

Compared to semantic distance approaches, supervised creativity scoring models far outpace their predecessors in predictive validity—often achieving response-level Pearson correlation coefficients with human ratings higher than.7 (e.g., DiStefano et al., 2024) and, in some cases, in excess of.8 (Acar et al., 2024; Goecke et al., 2024; Luchini et al., 2023; Patterson et al., 2024). Supervised scoring methods have been developed for a range of visual creativity tasks, such as the Multi-Trial Creative Ideation task (Acar et al., 2024; Patterson et al., 2024) and the Test of Creative Thinking—Drawing Production task (Acar et al., 2024; Cropley & Marrone, 2022). A similarly diverse set of scoring models has been developed for verbal creativity tasks, spanning the AUT (Organisciak et al., 2023), metaphor generation (DiStefano et al., 2024), short story writing (Luchini et al., 2024), scientific creative thinking (Beaty et al., 2024), and creative problem solving (Luchini et al., 2023), among others.

The recent explosion of testing and scoring software has made collecting and scoring creativity data easier than ever before. Yet, ‘easier than ever before,’ should not be taken to mean that collection and scoring is *easy*. For testing, the available software (e.g., PsychoPy) is study-general and not ready-made for creativity testing. While not necessarily an issue for individuals with expertise in experimental design and creativity assessment, for those without such expertise, the aforementioned expertise requirements are likely experienced as insurmountable barriers. For creativity scoring, the story is similar. Most of the available scoring models (e.g., convolutional neural networks) require some degree of programming skills as well as the ability to appropriately install the array of supporting software required to apply scoring models to creativity data.

Notably, however, some scoring models such as Ocsai and Ocsai-D (Acar et al., 2024; Organisciak et al., 2023), SemDis (Beaty & Johnson, 2021), and TransDis (i.e., a variant of SemDis designed for Chinese; Yang et al., 2024) offer cloud-based scoring via a graphical user interface (GUI). These point-and-click interfaces allow users to upload a CSV file containing creativity responses from anywhere in the world and download a CSV output file containing creativity ratings. Such interfaces have the desirable characteristic of making creativity scoring approachable for individuals of all levels of technical expertise, including those with no or minimal expertise. Yet, presently, only two cutting-edge scoring models (i.e., Ocsai/Ocsai-D) are available in GUI format.

One final consideration for why present software is not *easy* for individuals without expertise is that the different aspects of creativity measurement (i.e., testing and scoring) are decentralized. That is, one set of software tools is used to build the creativity measure, administer it to the desired sample, and process the collected data into a scorable spreadsheet file (e.g., CSV), while another set of software tools is used to score the collected data. While the work of implementing a creativity measure, distributing it to subjects, processing the data, and scoring the data represents commonplace and acceptable labor among the population of researchers, for others, the time, expertise, and labor needed to develop a complete pipeline—using decentralized tools—can make existing solutions impractical and unattainable for individuals who have limited time for such activities (e.g., educators).

To summarize, creativity is an essential 21st-century skill—and accurately measuring creativity is a vital step in helping educators assess the effectiveness of interventions intended to promote creativity. Currently, separate tools have been developed that make collecting and scoring creativity measures easier than ever before. Yet, extant tools still (1) require a high degree of domain-specific expertise to use (e.g., expertise in experimental design or translating creativity measures from primary literature to practice) and (2) require significant time and labor investments to establish and use a creativity measurement pipeline—in part due to the decentralized nature of existing testing and scoring tools. Accordingly, there is a significant need for solutions that democratize creativity measurement by making technological innovations freely accessible that enable non-experts to construct creativity assessments, collect data, and score assessments with limited time and labor investments. In the present work, we introduce the Creativity Assessment Platform (CAP), which aims to fill this need.

## Introducing CAP

CAP is a *free-to-use* web application^1^ for easily building, administering, and automatically scoring creativity measures (cap.ist.psu.edu). We believe that CAP is best experienced firsthand; we encourage readers to first explore the app, or to do so while reading through this manuscript. *Ease of use* is one of the guiding principles in CAP’s design (Table 1). In service of this goal, CAP’s interface is entirely GUI-based. In other words, the entire application is ‘point-and-click’—allowing users to build and score creativity assessments using only a mouse and keyboard, without requiring any programming knowledge and skills. CAP’s *ease-of-use* is further bolstered by not requiring skills in experiment design or translating measures from the creativity literature to a computerized assessment. All of the creativity tasks hosted on CAP are empirically validated and pre-packaged—enabling the user to straightforwardly select which tasks and items they wish to include in their creativity assessment, without the risk of introducing methodological errors to the assessment. For these reasons, CAP is easily approachable by seasoned creativity researchers and novices alike.

**Table 1:** Core principles guiding CAP development

Core principle	Description
Ease of Use	CAP is designed to be user-friendly with a point-and-click interface, requiring no programming knowledge to build and score creativity assessments.
Flexibility	While maintaining ease of use, CAP allows customization of tasks, items, and trial parameters to suit specific research needs.
Centralization	CAP integrates all aspects of creativity assessment, from task creation to data collection and scoring, within a single platform.
Empirical Validation	The tasks and scoring models provided on CAP are empirically validated, ensuring reliable and valid measurements of creativity.
Accessibility	CAP is free to use and accessible to educators and researchers at all levels, democratizing the measurement of creativity.
Multilingual capability	CAP includes multilingual scoring models for certain tasks, supporting the assessment of creativity across different languages.
Interoperability	CAP can connect with other platforms (e.g., Prolific, Sona) and software (e.g., Qualtrics) to expand its functionality within existing research pipelines.
Transparency and Reproducibility	CAP promotes research transparency and reproducibility by automatically generating method sections with key study details for journal articles.

Although *ease of use* can be at odds with *flexibility*, CAP is designed to provide a considerable degree of flexibility in study design—allowing users to customize the tasks, items, and trial parameters of their creativity assessments. CAP’s tasks span three domains of creative thinking: (1) Science, Technology, Engineering, and Mathematics (i.e., STEM), (2) verbal creativity, and (3) visual creativity. Supplementing CAP’s flexibility is the fact that CAP is interoperable with other data collection platforms (e.g., Prolific, www.prolific.com; Sona: https://www.sona-systems.com) and study design software (e.g., Qualtrics: Provo, UT). Thus, although CAP is limited to creativity assessment, researchers or educators can readily connect CAP-based studies to measures/experiments created on other platforms. Finally, *centralization* is a core tenet of CAP. CAP allows one to construct different creativity assessments, collect data, monitor data collection, and automatically score those data using empirically validated machine learning models—all within the same web tool. In other words, CAP provides an end-to-end creativity measurement pipeline—obviating the need to work with decentralized tools or spend time developing a pipeline that connects them.

The CAP web app is composed of three core sections: Creativity Metrics, Study Builder, and Task Playground. CAP is a more fully featured successor to the SemDis web application introduced by Beaty and Johnson (2021), which was limited to computing semantic distance and was not capable of testing/data collection. CAP’s Creativity Metrics section contains an array of creativity scoring models tailored for different creativity measures—including both legacy semantic-distance-based models (as offered on the SemDis web app) and contemporary supervised models (e.g., convolutional neural networks and large language models trained to match human creativity ratings). The Creativity Metrics Section is catered more to researchers who have already collected data and desire to use CAP solely for scoring. Users can upload CSV files and receive creativity scores from a model that is tailored to a particular creativity measure, such as the Alternate Uses Task.

The Study Builder section of CAP enables users to easily design creativity assessments via a simple point-and-click interface. After creating an assessment, a distributable study link is generated for data collection. The Study Builder section also comes with a User Dashboard that shows: all of the studies the user has created, the corresponding study links, and data monitoring variables (e.g., number of participants who have completed the study). Importantly, the User Dashboard also comes with autoscoring functionality; when data collection for an assessment has concluded, users can toggle this option to automatically score all of the data using empirically validated machine learning models tailored to each task in the assessment—no data preparation necessary. The Study Builder section of the app is designed to accommodate seasoned creativity researchers and novices alike. Finally, the Task Playground is a convenient way to sample the creativity tasks on CAP, from the participant’s viewpoint, without having to design and run a full creativity assessment.

In the following sections, we detail the functionality of the Study Builder and Creativity Metrics sections of CAP. As the Task Playground section recapitulates the tasks available via Study Builder and is merely intended for illustrative purposes, we omit further attention below.

## Study builder

The Study Builder functionality of CAP is designed to provide researchers and educators the tools they need to quickly and easily generate creativity assessments using a simple GUI, monitor data collection, and automatically score data via the User Dashboard. As creativity measures are central to constructing assessments via Study Builder, we will first detail the measures available on CAP before demoing the functionality and flexibility of Study Builder—from the design of creativity assessments to their autoscoring.

## Creativity measures

CAP offers empirically validated creativity tasks that span three domains of creative thinking: STEM creativity, verbal creativity, and visual creativity (Table 2). In the STEM domain, CAP currently offers (1) the Design Problems Task (e.g., Hu et al., 2022; Shealy & Gero, 2019) and (2) the Scientific Creativity Task (Beaty et al., 2024). In the Design Problems task, respondents are provided with one or more real-world scenarios that come from the following categories: Social and Environmental (e.g., *Develop as many design ideas as you can to reduce air pollution in cities*), Ability Differences and Limitation (e.g., *Develop as many design ideas as you can to assist people with learning impairments retain information*), and Transportation and Mobility (e.g., *Develop as many design ideas as you can to reduce the risk of accidents caused by distracted driving*). Participants are then asked to generate original and effective design ideas that could serve as solutions to the provided problem(s). Currently, CAP provides 15 Design Problems Task items: six in each of the Social and Environmental and Transportation and Mobility categories, and five in the Ability Differences and Limitation category. In each task, participants respond using their keyboard or mouse, and can edit their responses before submission using arrow keys, and the backspace/delete keys. Participant responses are stored in the CAP database under the user’s account ID.

**Table 2:** Summary of CAP domains, tasks, items, and scoring models

Domain	Task	Example items	Scoring model
STEM creativity	Design Problems Task	“Develop as many design ideas as you can to reduce air pollution in cities”	AIDE (Artificial Intelligence for Design Evaluation)
	Scientific Creativity Task	“What scientific questions could you ask about a robot that can learn and think like humans?”	SCTT-AI (Scientific Creative Thinking Task AI)
Verbal creativity	Alternate Uses Task (AUT)	“Come up with alternate uses for a BRICK.”	CLAUS (Cross-Lingual Alternate Uses Scoring)
	Short Story Task	“Write a 4-5 sentence story that incorporates the words Belief-Faith-Sing.”	MAoSS (Multilingual Assessment of Short Stories)
Visual creativity	Drawing Task	“Use your mouse to make a drawing that incorporates the shape.”	AuDrA (Automated Drawing Assessment)

In the Scientific Creative Thinking Test (SCTT; Beaty et al., 2024), respondents are provided with hypothetical scenarios and are asked to respond by generating one of three categories of response that would facilitate scientific understanding of the scenario: research questions (e.g., *You are introduced to a robot that can learn and think like humans. What scientific questions could you ask about this?*), hypotheses (e.g., *On a field trip, you drive past a massive field with hundreds of large holes visible as far as the eye can see. What hypotheses do you have about what purpose the holes may serve?*), or experiment design/operationalization (e.g., *You think students at your school are friendlier than students at most schools. How could you test that hypothesis?*). The SCTT has been shown to exhibit high reliability and external validity (Beaty et al., 2024). Users of CAP can choose from 15 Scientific Creativity Task items, five items in each of the research questions, hypotheses, and experiment design categories.

In the verbal creativity domain, CAP currently offers two measures: the Alternate Uses Task (AUT: Guilford, 1967) and the Short Story Task (Johnson et al., 2023; Prabhakaran et al., 2014). In the AUT, participants are provided with a prompt/item object (e.g., *brick*) and are asked to generate unusual or creative ways the object could be used. CAP provides 15 AUT prompts/items to choose from. In the Short Story Task, participants are presented with three prompt words (e.g., *belief-faith-sing*) and the respondent is asked to generate one or more five-sentence short stories that contain all three prompt words. Users can pick from six three-word prompts for the Short Story Task. Both verbal tasks on CAP have been used extensively in the creativity literature and have received ample support for their reliability and validity (e.g., Benedek et al., 2013; Forthmann et al., 2021; Prabhakaran et al., 2014).

Finally, to assess visual creativity, CAP offers the Drawing Task. The Drawing Task is based on the ‘incomplete shapes’ task from Barbot’s (2018) Multi-Trial Creative Ideation measure. In the Drawing Task, participants are provided with a starting image consisting of four contours that loosely exhibit Gestalt closure. Participants then sketch a drawing that is as creative as possible, incorporating the starting contours. To complete Drawing Task trials, participants draw lines and/or contours on the starting image with their mouse; an eraser tool and an ‘Undo Last’ button allow participants to remove accidental or undesirable mouse strokes on the drawing canvas. Additionally, a text input box below the drawing canvas prompts participants for a description of what they drew (“*What did you draw?*”).

## Study builder walkthrough

**Fig. 1:**
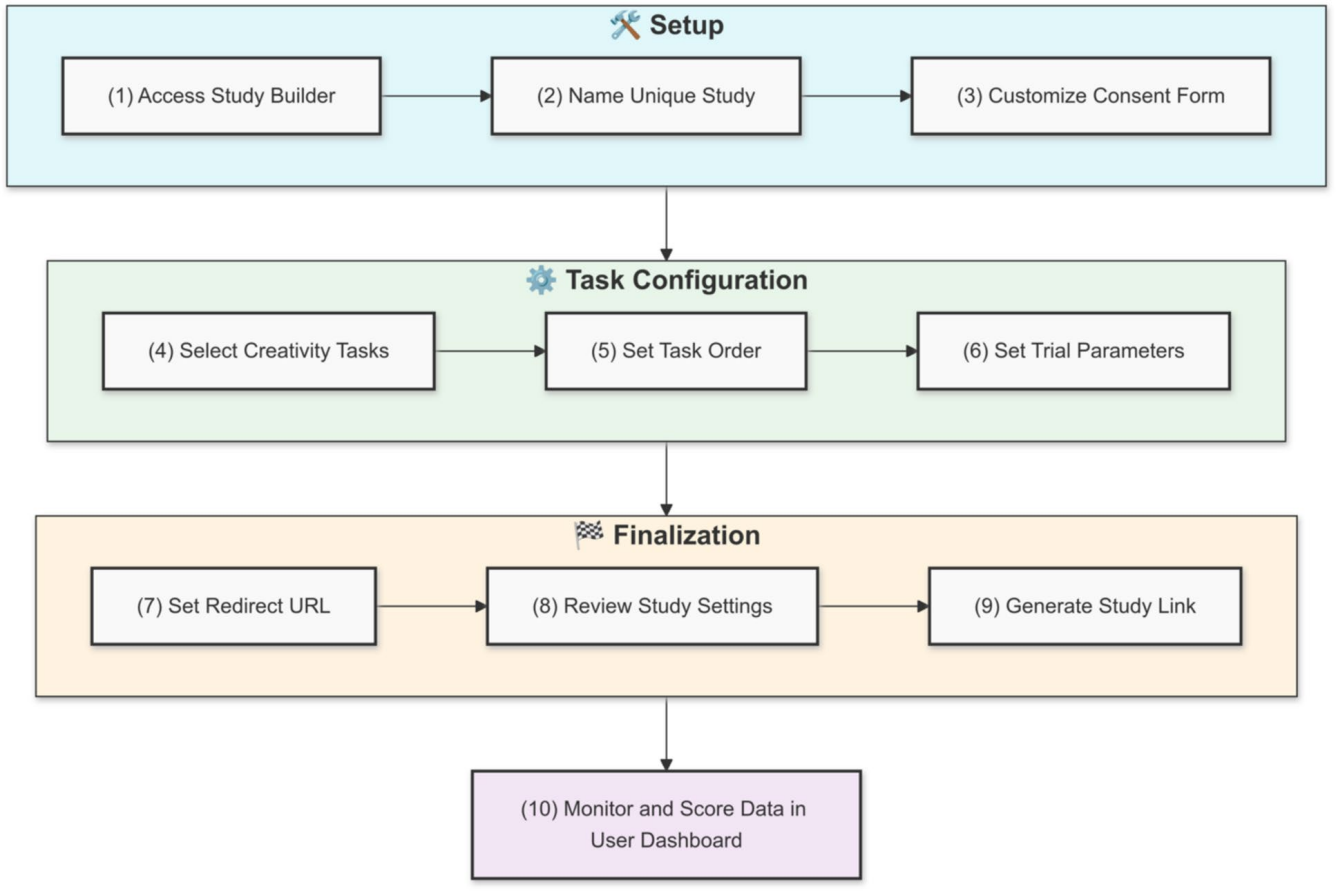
Workflow of study builder

To use Study Builder, the user must first register an account on the CAP website. This is because generated studies must be associated with individual users in the CAP database. CAP is free for researchers and educators. Users can request an account by entering a valid school- or research-affiliated e-mail address, their institution’s name, a link to their faculty page or staff directory, as well as a desired username and password. At this point, users must agree to the terms and conditions: “By using the Creativity Assessment Platform, you are responsible for obtaining necessary research ethics approvals for storing potentially personally identifiable data. We provide temporary data storage up to 30 days for your access, but long-term storage is your responsibility. We encourage you to download your data from the website once your study is complete.” The user must also specify if they would like to opt-in to sharing the deidentified data collected via their account, which will be used to improve CAP’s scoring models and gain insights that improve CAP’s offerings. After confirming the user’s details CAP administrators will approve the account registration, at which point the user can log in and gain access to the Customize Tasks and User Dashboard functions, found under the Study Builder dropdown. To create a custom experiment, users simply click the Customize Tasks link. Once clicked, the user is presented with a page that describes what Study Builder offers along with a button to ‘create your study’. Once the ‘create your study’ button is clicked, users are walked through a series of pages to customize their study/assessment. The flow of Study Builder is portrayed in Fig. 1.

The first point of customization is selecting a name that will uniquely identify the study; this study name will appear in the User Dashboard, where the user can monitor data collection progress and access other functionality (detailed further below). On the following page, users can select which language they wish their creativity assessment to be presented in; CAP currently offers ten languages (English, Chinese [Mandarin], Dutch, French, Farsi, German, Italian, Polish, Russian, and Spanish). On the next screen, users can optionally enter the informed consent form they want participants to read at the beginning of the study. The following page presents users with options for a basic demographics survey at the beginning of their study, after informed consent; users can select any combination of age, gender, and ethnicity, or none at all to omit the demographics survey altogether. On the next Customize Tasks page, users can select which tasks they wish to include in their creativity assessment. On the following page, users can select the order in which they would like their creativity tasks to appear when participants complete the study/assessment (or optionally select that each participant will experience a random task order).

The next Customize Tasks page allows users to select trial-level parameters, including: (1) how long participants have to respond to each stimulus/item and (2) how many responses the participant must submit for each stimulus. Both trial duration and number of required responses are specified in integers, with duration corresponding to seconds. Users can specify that trials have unlimited time or an unlimited number of responses (but not both) by entering −1 in the input box. After the trial parameters are specified, on the next page of Customize Tasks, users can customize the general task instructions for the creativity tasks they have chosen for their study. The task instruction customization page comes prepopulated with default, literature-supported instructions for each task—removing the burden of generating instructions for those who are unfamiliar with creativity assessment. While instruction customization is not necessarily encouraged, CAP provides flexibility to researchers who wish to explore the effects of instructional manipulations (e.g., ‘be creative’ vs. ‘be fluent’; Forthmann et al., 2016).

The next *n* pages—where *n* corresponds to the number of tasks the user selected to include in their study—allow users to pick which items from each creativity task they wish to include in their study. Note: users can only select from the provided items and not edit or add custom items. This is important for ensuring that the AI scoring models will be able to accurately score participant responses. Following the item specification pages, the next page of Customize Tasks allows the user to specify where (if anywhere) they would like the participants to be redirected to after completing the CAP-designed study. This redirect functionality allows CAP to be interoperable with other platforms such as Qualtrics, Pavlovia, Sona, or Prolific; note that credit codes can be included in a query string at the end of the redirect URL to grant the participant credit/payment upon completion of the study in cases where services like Sona or Prolific are part of the user’s study pipeline (e.g., ‘sona-systems.com/webstudy_credit.aspx?experiment_id=YOUR_EXPERIMENT_ID&credit_token=YOUR_CREDIT_TOKEN&survey_code=’, where CAP will automatically append the participant ID at the end and prepend ‘https://’ to the beginning). On the next page of Customize Tasks, the user gets a ‘receipt’ showing the options selected for the study. This page represents the final chance the user has to go back (using the ‘Back’ buttons present on each of the preceding pages) and make edits to their study; once they click ‘Next’, the study will be finalized. Finally, after the user clicks ‘Next’, the app will store all of the selected options in the CAP database (under the user’s ID) and a study link will be generated that can be distributed to participants.

**Fig. 2:**
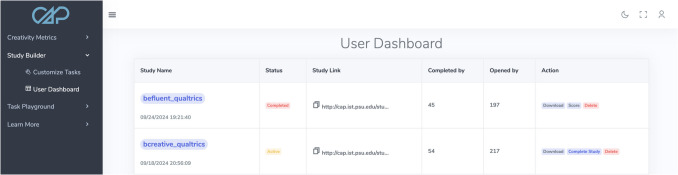
CAP researcher dashboard

A key facet of CAP that makes it suitable for those with little-to-no experience with creativity research is the User Dashboard page (see Fig. 2). The User Dashboard is a centralized interface from which study creators can (1) examine details of the created study, (2) monitor data collection, (3) download collected data, and (4) autoscore all collected data from a given study with a click of the mouse. Upon designing a creativity study via the Customize Tasks pipeline in Study Builder, an entry is created in the User Dashboard table. The *Study Name* column of User Dashboard recapitulates all of the core details of the created study: when the study was created, the study’s name, the tasks and item IDs the user selected for each task, as well as how many responses participants are allowed to make per item and how long they have to submit their responses to each item. The *Status* column identifies whether the study is currently active or not (more on how this is toggled follows below). The *Study Link* column contains the link to the generated study with a convenient copy-to-clipboard button. For keeping track of the study’s progress, the *Opened by* and *Completed by* columns identify how many participants have begun the experiment (i.e., clicked the study link) and finished the experiment, respectively.

The final column of the User Dashboard is called *Action*. The *Action* column features three buttons: ‘Download’, ‘Complete Study’, and ‘Delete’. As implied by the name, the ‘Download’ button allows users to download their raw *unscored* study data along with an automatically generated method section. The raw data download functionality was included so that users could check on data quality during collection; users can also score the raw responses manually if they so choose, or upload them to Creativity Metrics to score them at a later date. The automatically generated method section includes the core study details available under the *Study Name* column of User Dashboard in prose format with helpful citations. While oriented more toward researchers rather than educators, the ‘auto-method’ functionality offered by the ‘Download’ button was included to standardize reporting of tasks/autoscoring parameters and make it easier to disseminate research conducted using CAP. For example, users can copy/paste the auto-method text into the Methods section of a manuscript.

The ‘Complete Study’ button serves two purposes: (1) to close out a study permanently (which initiates a 30-day countdown to data deletion and marks the study as complete in the *Status* column) and, after doing so, (2) a ‘Score’ button appears that grants access to autoscoring of the study’s dataset^2^. Once the ‘Score’ button is clicked, each task in the user’s study is scored using the most performant scoring model for that task; the scored data is then downloaded to the user’s computer along with the auto-method section that includes details about the tasks, scoring models, and relevant citations.

Before continuing on, a point should be made about the security of CAP’s server and collected data. The CAP server is housed at The Pennsylvania State University, in the Department of Information Systems Technology’s server center. As such, the web app and the server itself adhere to Penn State cybersecurity standards. During the development of CAP, our team consulted with cybersecurity specialists at Penn State; CAP was constructed according to OWASP security standards. After development, CAP was tested and passed a required stress test by university cybersecurity specialists aimed at uncovering potential security flaws. The CAP server is managed by information technology professionals who keep tabs on emerging cybersecurity threats and make necessary software updates to defend against those threats. While every reasonable step to ensure the security of CAP and users’ data has been taken, the risk cannot be entirely eliminated. Data collected via CAP will not be stored past the 30-day deletion window (after a study has been marked complete) and will not be shared—unless the user has opted into data sharing upon account creation. In such cases, the data will be shared only with the CAP team in order to improve scoring models and generate insights to augment CAP’s offerings.

In the next section, we will detail the different scoring models available on CAP, which are accessible via the Creativity Metrics section.

## Creativity metrics

The Creativity Metrics section of CAP houses all of the empirically validated models used to autoscore the creativity measures on CAP. The Creativity Metrics section allows users to upload a UTF-8 CSV file to a model tailored to a particular creativity measure, and receive a CSV output file that contains all the original columns plus new columns with creativity scores and a model identifier—all without making an account on CAP; uploaded data are immediately deleted from the CAP server after scores have been returned to the user. Although users who create a study on CAP are able to download their study data, separate it by task, and obtain scores by submitting each task’s data to the relevant scoring models, we anticipate that the Creativity Metrics section will primarily be of interest to researchers/users who have designed their experiment and collected data using another platform but wish to reap the benefits of autoscoring using a simple point-and-click interface. As noted above, although many open-source creativity scoring models exist, most of them require complicated software installation and some degree of programming knowledge to use. Accordingly, the Creativity Metrics on CAP provide an approachable solution for users who desire autoscoring but who wish to avoid unnecessary software complexity. Below, we will describe each model’s purpose as well as how each can be used.

## Semantic-distance models (unsupervised)

As mentioned above, CAP is intended to be a more fully-featured successor to the SemDis web app (semdis.wlu.psu.edu). The SemDis web app featured two scoring models: SemDis and DSI. Although the SemDis model can be applied to different tasks, it is typically used to provide semantic distance scores between an AUT prompt and all of the words in an AUT response—where greater distance between the prompt and all of the words in the response corresponds to more remote associations, which correlates positively with human creativity ratings. DSI, on the other hand, measures the average semantic distance between all pairs of words in a passage, where greater average distance indicates a greater amount of remote associations in the passage (which is positively correlated with human creativity ratings). Although DSI can be applied to any passage-form data, it is most extensively validated using the Short Stories Task (see Johnson et al., 2023).

Like the SemDis web app, the Creativity Metrics section of CAP provides access to both SemDis and DSI. However, while the DSI metric exactly matches that available on the SemDis web app, the SemDis measure available on CAP reflects recent innovations that have taken place in the literature. Specifically, the version of SemDis on the SemDis web app (1) used older, ‘bag-of-words’ word vector embeddings to represent words in a response and (2) was limited only to responses generated in English. Regarding (1), bag-of-words models (e.g., LSA, word2vec) can represent language reasonably well, but have no mechanism to alter word meaning based upon context. This prohibits the model from handling cases of connotation and polysemy (e.g., *ban*k in the money versus river sense). Recent advances in natural language processing, however, have yielded the transformer (Vaswani et al., 2017), which can alter word representations based on context. Word vector representations from a transformer markedly improve the degree to which semantic distance scores correlate with human creativity ratings (e.g., Johnson et al., 2023). Regarding (2), myriad multilingual transformers have been developed (e.g., Conneau et al., 2020); Wu & Drezde, 2019). Capitalizing on both advances, Patterson, Merseal et al. (2023) developed a new multilingual transformer version of SemDis that accommodates 12 languages: Arabic, Chinese (Mandarin), Dutch, English, French, Farsi, German, Hebrew, Italian, Polish, Russian, and Spanish. While not as accurate as the supervised models detailed below, this version of SemDis achieves small to moderate Pearson correlations with human creativity ratings at the response level, depending on the language (*r* =.23 to.52; Patterson, Merseal et al., 2023). DSI is similarly outpaced by its supervised counterpart below, but achieves moderate to strong correlations with human creativity ratings at the person aggregation level (*r* =.35 to.77; Johnson et al., 2023). Both the contemporary version of SemDis and the original DSI are accessible via the Creativity Metrics section of CAP.

**Fig. 3:**
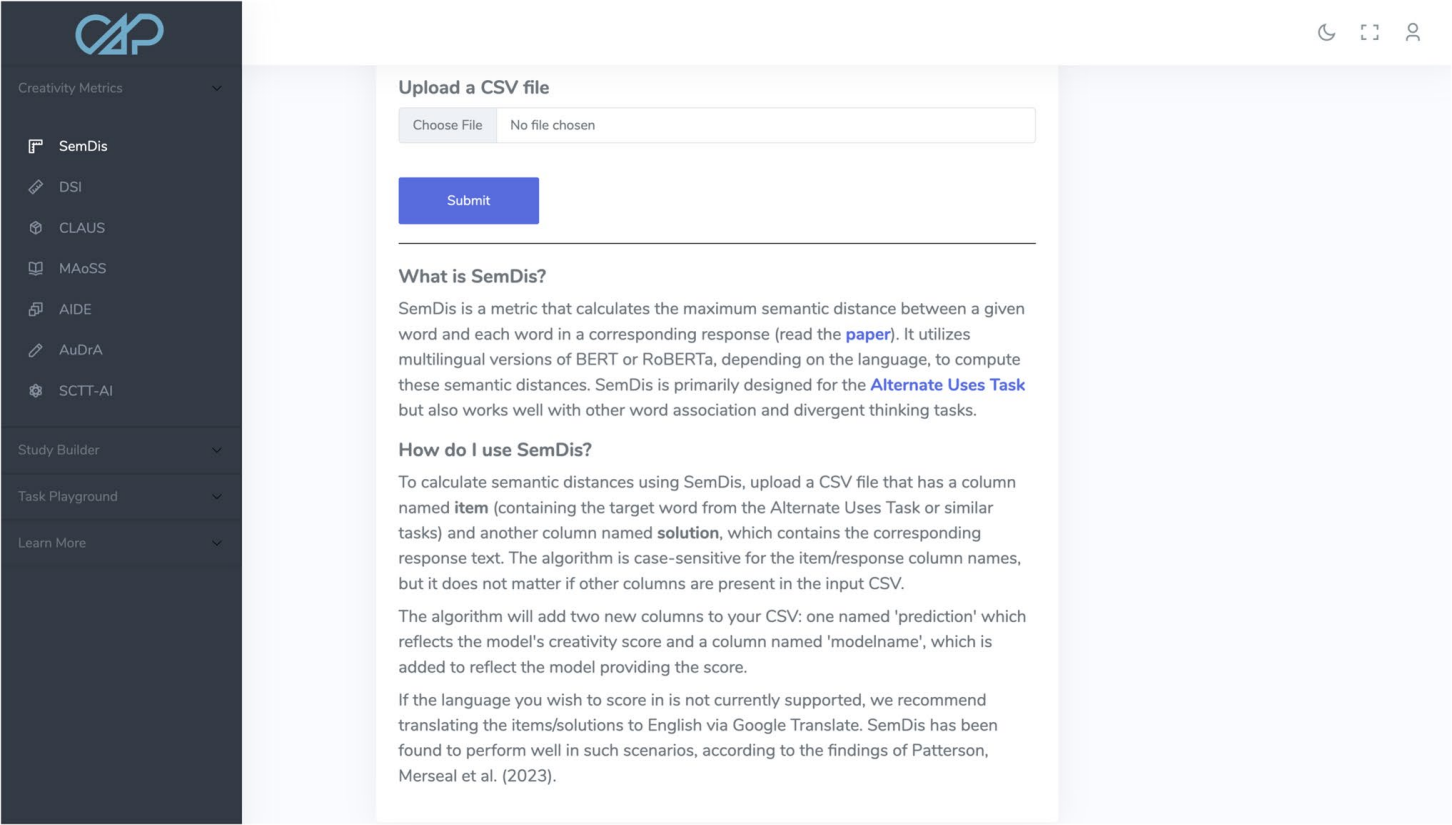
Illustration of the SemDis page of CAP’s Creativity Metrics section

To use the SemDis model, the user first needs to collect their data in a CSV spreadsheet that contains at least two columns: ‘item’, which contains the AUT or reference item against which all of the words in the response will be compared, and ‘response’, which contains the response. Both columns should be formatted as text. With their data formatted, users can navigate to the SemDis page, under Creativity metrics (Fig. 3), and click ‘Choose File.’ This will allow the user to search their computer’s file system for the target CSV file. Once selected, the user simply clicks ‘Submit’ and CAP will begin calculating semantic distance scores for each response/row in the CSV. After submitting, a progress bar will appear to give users a sense for how long scoring will take. After the algorithm has completed scoring, it will download a CSV to the user’s computer containing all the original columns, plus two new ones: ‘prediction’, which contains semantic distance scores, and ‘modelname’, which specifies that SemDis was used for scoring. The procedure for using DSI is identical to that of SemDis, except only a ‘response’ column—containing passage-form responses—is required for the uploaded CSV, and the scored CSV will show ‘DSI’ in the ‘modelname’ column.

## Supervised models

CAP is also home to five cutting-edge neural network models that have been trained to predict creativity ratings. For terminological clarity, by trained here, and throughout the manuscript, we mean pre-trained models that we have fine-tuned with additional data. Each model is tailored to a specific creativity measure—one model for each of the five creativity measures currently available on CAP. Below, we will detail each model, its intended purpose, and how to use each on CAP.

## CLAUS

CLAUS—the Cross-Lingual Alternate Uses Scoring model—is a multilingual transformer that has undergone supervised training to score responses from the AUT. CLAUS has been empirically validated to accurately (*r* =.65 to.77 at the response-level, depending on language) predict the creativity of AUT responses it was never trained on in ten different languages: Chinese (Mandarin), Dutch, English, French, Farsi, German, Italian, Polish, Russian, and Spanish. Appendix Fig. 4 reports the model training and validation of CLAUS based on 136,621 AUT responses and human ratings. As CLAUS substantially outperforms SemDis in predicting human creativity ratings, the autoscoring functionality of User Dashboard employs CLAUS by default when scoring a creativity assessment includes the AUT. The procedure for using CLAUS to get AUT creativity scores is identical to that of SemDis: the user needs to ensure their data is in a CSV spreadsheet with an ‘item’ column, corresponding to the AUT items (e.g., brick, paperclip), and a ‘response’ column which contains AUT responses. Users can then click the ‘Choose File’ button to search their computer’s file system for the to-be-scored CSV and click ‘Submit’ to begin scoring. A progress bar will show the proportion of responses that have been scored—to give the user a sense for how much time scoring will take and, after scoring is completed, the original CSV is returned along with ‘prediction’ and ‘modelname’ columns that contain creativity scores and the model used to score the items (i.e., CLAUS).

### MAoSS

The Multilingual Assessment of Short Stories (MAoSS) model is a multilingual transformer that has been trained specifically on responses to the Short Stories Task, and their corresponding human creativity ratings. Luchini et al., (under review; 2024) validated MAoSS as a Short Stories scoring model—showcasing human-model Pearson correlations ranging from.73 to.86, depending on the language. MAoSS is designed to accommodate Short Stories Task responses in 11 different languages: Arabic, Chinese (Mandarin), Dutch, English, French, Farsi, German, Hebrew, Italian, Polish, Russian, and Spanish. Using MAoSS via Creativity Metrics on CAP requires as input a CSV with two columns named ‘item’ and ‘response’, corresponding to the three-word Short Story prompts (e.g., stamp-letter-send) and the generated short stories, respectively. Everything else, from uploading the CSV from the user’s file system to downloading the updated CSV to the user’s computer, is the same as SemDis—with the exception that the ‘modelname’ column will identify the model as MAoSS.

### AIDE

The Artificial Intelligence for Design Evaluation (AIDE) model is tailored to the Design Problems Task on CAP. Like the supervised models above, AIDE was trained to predict the creativity of solutions to the Design Problems Task. Unlike the above models, however, AIDE has additionally been trained to predict human-provided ‘effectiveness’ scores, which provide a measure of how effective the proposed solution is for solving the problem scenario. Empirical work (Appendix Fig. 5) has demonstrated that AIDE provides highly accurate predictions that closely match human-provided ratings for creativity (*r* =.78) and effectiveness (*r* =.63) on responses the model was never trained on. AIDE is currently available in English only. To use AIDE, the user needs to prepare a CSV containing at least two columns named ‘item’ and ‘response’, which contain the Design Problems Task items and participants’ responses, respectively. After selecting and submitting the CSV to AIDE, a progress bar will indicate how far along scoring is and, once complete, an updated CSV will be downloaded to the user’s computer with three new columns: ‘prediction’, ‘effectiveness_prediction’, and ‘modelname’, which contain the creativity score, effectiveness score, and the model identifier, respectively.

### AuDrA

The Automated Drawing Assessment (AuDrA) model is a convolutional neural network trained to predict human creativity scores on the Drawing Task. AuDrA was first introduced in Patterson et al. (2023) as a solution for scoring Barbot’s (2018) drawing task. AuDrA was shown to achieve high accuracy in predicting human ratings for drawings it was never trained on (*r* =.81). For CAP, we introduce a new version of AuDrA that is equally as performant on untrained drawings, but has the added capability of rejecting scribbles and other off-task responses (Appendix C). To use AuDrA on CAP, users simply need to navigate to the AuDrA tab under Creativity Metrics and click ‘Choose Files’. The user can then use their file system to select drawings from the CAP Drawing Task or Barbot’s (2018) Multi-Trial Creative Ideation task and click ‘Submit’ to begin scoring. Note: uploaded drawings will be deleted from the CAP server following scoring completion. Like the other models, AuDrA will indicate scoring progress via a visual progress bar. Once scoring is complete, a CSV will be downloaded to the user’s computer containing two columns: ‘filenames’ and ‘predictions’, which contain the names of the image files uploaded to CAP and their associated creativity scores.

### SCTT-AI

The Scientific Creative Thinking Task Artificial Intelligence (SCTT-AI) model is a transformer that has been trained to predict human creativity ratings corresponding to Scientific Creativity Task responses. The trained SCTT-AI model has been empirically shown to achieve high degrees of accuracy in matching human creativity ratings for responses the model was never trained on (*r* =.72; Beaty et al., 2024). To use SCTT-AI, users need to upload a CSV containing two columns: ‘item’ and ‘response’, which contain the Scientific Creativity Task items/questions and participants’ responses, respectively. Like the other models, scoring progress will be shown via a visual progress bar and, after scoring completes, a CSV will be downloaded to the user’s computer which contains two additional columns: ‘prediction’ and ‘modelname’, which stores SCTT-AI’s creativity predictions and the model identifier, respectively.

## Discussion

Creativity is a vital 21st-century skill, and accurately and efficiently measuring creativity will be essential to fostering creativity within education and beyond. To achieve this goal, we introduce CAP—a user-friendly web application for designing, administering, and scoring creativity assessments in the present work. We believe that CAP is uniquely poised to make an impact on creativity measurement, both among creativity researchers and educators. For educators, the point-and-click simplicity of the interface, pre-programmed creativity measures, and easy autoscoring functionality (no data preparation required) make for a tool that can be readily adopted with very little startup cost. For seasoned creativity researchers, we anticipate the models available via the Creativity Metrics section of CAP will be of primary interest, especially among those who have their own data collection pipelines but wish to speed the pace of research by automating scoring. However, we also expect that Study Builder will be of interest for researchers who either do not have an established data collection pipeline or who wish to incorporate CAP’s creativity assessments into their existing data collection pipeline.

While previous software innovations have made it easier than ever before for *researchers* to build creativity studies and automatically score their data, creating and scoring creativity assessments still requires a high degree of expertise (e.g., experimental design, programming) along with considerable time and labor investments. Reducing those barriers to entry—so that anyone can easily design, administer, and score creativity assessments—was a central motivation for building CAP. It is our view that, by virtue of its centralization and ease of use, CAP substantially lowers, or altogether eliminates, those barriers to entry. Considering that CAP is a free-to-use web app, we are hopeful CAP will promote the democratization and proliferation of creativity assessment both within research and education.

While CAP makes substantial inroads toward making creativity assessment accessible to everyone, it is important to note limitations of the platform and, where applicable, planned further development that is aimed at addressing those limitations. First, CAP is designed to make constructing creativity assessments a simplistic process. While we believe CAP accomplishes this goal, this goal is at odds with flexibility in study design. Users cannot create arbitrarily complex studies or make arbitrary changes to CAP measures, such as generating custom items; this was done in part with the aim of limiting CAP to *validated* measures and items. Instead, study creation on CAP is constrained to the tasks/items included on the platform, with a limited set of customizations available (e.g., task instructions, task order, trial duration, trial response allowance). While constraining the space makes for a more approachable experience—especially for creativity measurement novices—researchers may find the limited pallet of tasks and customizability confining in some cases. In cases where further customization is desired, we encourage researchers to either (1) design custom versions of CAP’s tasks on other platforms (e.g., PsychoJS, lab.js) and use CAP’s Creativity Metrics to score said tasks or (2) if researchers want to use *some* of CAP’s tasks as-is but want to customize *other* CAP tasks, users can create custom versions of CAP tasks on another platform and redirect to or from CAP, using CAP’s redirect option in Study Builder. Relatedly, we also acknowledge that, while CAP covers three important domains of creativity (i.e., visual, verbal, and STEM), CAP currently only hosts five creativity measures (and corresponding fine-tuned scoring models) across those domains. While this is a modest number relative to the overall population of creativity measures that exists, we view CAP as a work in progress and aim to incorporate additional empirically validated measures for which there is sufficient data to train a scoring model.

Another limitation pertains to the languages supported by CAP. While three of CAP’s scoring models support multiple languages (i.e., SemDis, CLAUS, and MAoSS), the rest currently do not. Simultaneously, CAP offers the ability to present creativity experiments in ten languages. We conducted alpha testing on CAP with an international sample of researchers who tested CAP as part of their own research. A common refrain from testers was the desire to build creativity tests in their native language, even if the automatic scoring models do not yet support those languages. Although our original intent was to support only languages with associated scoring models, in the interest of democratizing creativity measurement, we have added support for all ten languages in Study Builder. Of course, our primary aim for CAP is to make it a closed-loop, end-to-end solution for creativity measurement. Accordingly, a chief goal for future work is to train multilingual variants of the remaining English-only scoring models. It should be noted that training such models requires thousands of human-rated responses per task; developing a novel model is a considerable undertaking, but our research team and international collaborators are committed to this goal and have already made substantial progress, as evidenced by the multilingual scoring models noted above (Luchini et al., 2024; Patterson et al., 2023) and the development of multilingual study administration. Additionally, we are hopeful that users will be willing to opt into the data sharing agreement at signup if it means better coverage for automatic scoring in the long term. In the interim, however, it should be noted that while not all of CAP’s models currently support multilingual scoring, recent research suggests that translating unsupported languages to English can yield comparable results relative to when the responses are submitted to a scoring model untranslated to a multilingual model (Zielińska et al., 2023).

In the interest of making CAP as broadly useful as possible—in addition to coverage of multiple languages—consideration should also be given to the type of respondent. A growing body of work has focused on AI creativity and human-AI hybrid creativity (e.g., Hitsuwari et al., 2023; Koivisto & Grassini, 2023; Medeiros et al., 2025). While the Creativity Metrics on CAP will readily provide scores to responses from AI and hybrid sources, it remains an open question how accurately the models would match human creativity judgments for those same responses. As the models were trained only on human responses and creativity ratings, there is a (substantial) risk that the language produced by AI is distributionally different from that of the humans used to train the models. To the extent this is true, CAP’s predictions may be inaccurate. Accordingly, it will be crucial to investigate potential model biases related to this or other factors in future work. In the event that biases are found, additional data collection and model retraining will be necessary to ensure CAP’s predictions are valid under as wide a variety of circumstances as possible.

One last limitation stems from CAP’s objective to make creativity measurement as accessible as possible. While CAP dramatically reduces expertise-tied barriers to collecting and scoring creativity measures, CAP currently does not have functionality targeted at *interpreting* scored creativity data. CAP’s autoscoring function provides users with responses scored at the response level. While this affords users with maximal flexibility as to how they analyze or plot the data, for those without experience aggregating data at the person or sample level (using spreadsheet software or statistical programming languages like R; R Core Team, 2020), the returned scores may be difficult to make sense of or act on. Educators, for instance, may be inclined to use CAP to measure the effect of in-class interventions designed to promote student- and/or class-level creativity. However, the scored response-level data that CAP returns does not directly permit inference-making in lieu of aggregation and/or analysis. As such, a key future direction for CAP is to implement a basic analysis tool that enables researchers and educators to automatically perform data aggregation, plotting, and statistical significance tests tailored to their specific interests and needs. Nevertheless, we hope this tool will benefit researchers and educators alike, and facilitate broad-based examination of creativity—as well as the factors that promote it.

CAP provides the first-ever infrastructure to unify cutting-edge creativity scoring models (which often require expertise to use) with a platform that enables users to easily build custom creativity assessments—all in one place. Through its ease of use and functionality, we believe CAP will empower creativity researchers and educators alike to advance our understanding of creativity and how to promote it.

## Figures and Tables

**Fig. 4:**
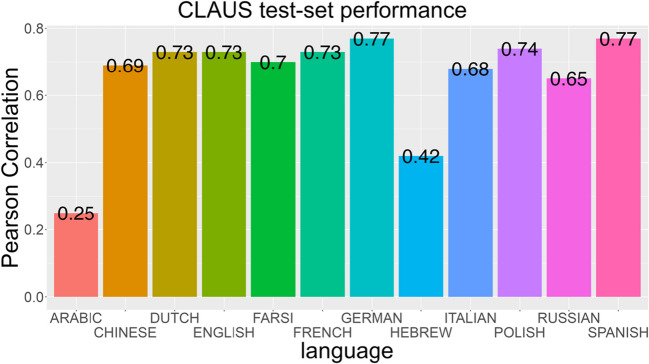
Pearson correlation by language on the held-out test set

**Fig. 5:**
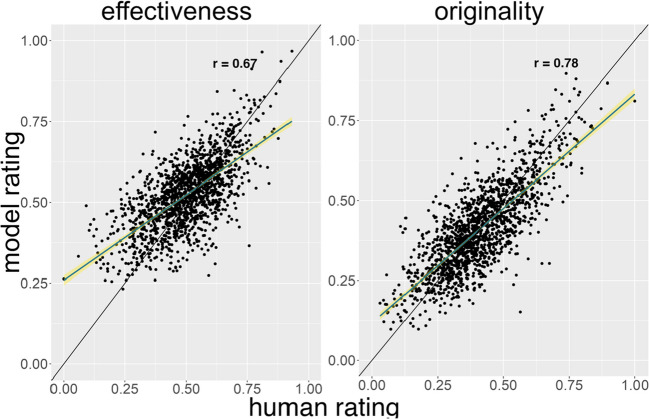
Scatter plot of model predictions as a function of human ratings for effectiveness (left) and originality (right)

## Data Availability

All data for empirical work in this manuscript are available on OSF (https://osf.io/4nkmj/).

## Appendix A Development and validation of the Cross-Lingual Alternate Uses Scoring (CLAUS) model

To develop the CLAUS model, we employed the large multilingual dataset gathered by Patterson, Merseal et al. (2023). The dataset consists of 107,672 AUT responses and human-provided creativity ratings in 12 different languages: Arabic, Chinese (Mandarin), Dutch, English, Farsi, French, German, Hebrew, Italian, Polish, Russian, and Spanish (see Patterson, Merseal et al., 2023 for distribution by language and additional details). We further supplemented this dataset with additional English AUT responses from Organisciak et al., (2023; *n* = 5490), Mohr et al. (2016; *n* = 3864), as well as in-house data from prior experiments (*n* = 19,595). On an experiment-wise basis, we then computed Judge Response Theory theta scores (Myszkowski & Storme, 2019) for each response using the human creativity ratings. Theta scores help adjust for rater severity and provide estimates of the underlying construct of creativity present within the responses. We have trained creativity scoring models with theta scores successfully in other work (e.g., Patterson et al., 2024). Finally, we removed exact duplicates from the dataset and split the data using a 70/10/20% split into training (data the model gets to learn from), validation (data used to judge which model settings best aid performance), and held-out test (data we use to evaluate how well the model generalized to untrained responses). The data splits were balanced by language—i.e., 70% of each language was used in the training set Figure 4.

We trained XLM-RoBERTa (‘xlm-roberta-base’ in the Huggingface transformers package, Wolf et al., 2020)—a lean but capable multilingual language model—on the training dataset using an elaborate task framing which always was presented in English, regardless of the language of the AUT item and response (“Identify how surprising, creative, unexpected, or interesting the following alternative use for the object <<OBJECT>> is: <<RESPONSE>>”). We evaluated different model hyperparameters (learning rate, number of training updates) using the validation set. The final version of the model used a learning rate of 5e^-5^ and was trained for 52,500 training steps using a batch size of 16. We then administered the held-out test set to judge the ability of the final model to generalize to untrained AUT responses. The model-predicted scores correlated strongly with human-provided creativity ratings (Figure A); most languages fell between *r* =.65 (Russian) and *r* =.77 (German) with only two of the 12 languages falling below the.65 level—Arabic and Hebrew. It should be noted that these two languages had the two fewest responses and, further, Arabic was the only dataset rated by only a single rater. Code, items, and scores are available on OSF.

## Appendix B Development and validation of the Artificial Intelligence for Design Evaluation (AIDE) model

AIDE was developed to score responses to the Design Problems task for both their degree of creativity as well as their effectiveness. We collected a dataset of 8477 responses that were scored by a set of 20 raters for both their degree of creativity and effectiveness (i.e., how well the proposed solution solves the design problem). Prior to training the model, we computed factor scores for each response using these ratings, which we then transformed to a 0–1 scale using min-max scaling. The dataset was then split 70/10/20% into training, validation, and held-out test subsets, respectively Figure 5.

We then subjected a pre-trained RoBERTa base (‘roberta-base’ on Huggingface, Wolf et al., 2020) to training on the training dataset and searched for which hyperparameter values best promoted model accuracy on the validation dataset—once based on human-provided creativity ratings and a second time based on human-provided effectiveness scores. We searched over learning rate, batch size, and number of training steps when training the creativity model to determine which hyperparameters minimized mean square error between human and model-predicted ratings on the validation set. We then transferred the best-fitting learning rate and batch size to the effectiveness model, but optimized for the number of training steps that minimized mean square error between human and model-predicted effectiveness ratings on the validation set. The most performant creativity model variant used a learning rate of 2.76e-6, a batch size of 16, and was trained for 4050 steps. The best-fitting effectiveness model (which used the same learning rate and batch size) achieved peak performance after 4613 training steps. We then subjected each best-fitting model to the held-out test set to determine how well each generalized to untrained design problem responses. We found both models’ predictions highly aligned with human-provided ratings of creativity (*r* =.78) and effectiveness (*r* =.63). A scatter plot of predicted versus actual ratings for each is shown in Figure B. Code, items, and scores are available on OSF.

## Appendix C Update of the Automated Drawing Assessment (AuDrA) model

In the work of Patterson et al. (2024), AuDrA was developed as a model to score drawings from Barbot’s (2018) Multi-Trial Creative Ideation (MTCI) task—an adaptation of which is used as the Drawing Task on CAP. In that work, the final version of AuDrA was shown to generalize strongly to drawings the model was not trained on (*r* =.81). Despite AuDrA’s strong performance, the model was only trained on *valid* responses to the MTCI—creating a risk that AuDrA might inaccurately assess responses that are invalid or off-task as more creative than they actually are. Accordingly, we collected a large batch of invalid responses (*n* = 7194), including scribbles and inappropriate drawings, and associated scores of 0 (the minimal value on the scale) with them. We then retrained AuDrA using the exact same hyperparameters judged to be best in Patterson et al. (2024) but using the invalid-augmented training, validation, and held-out test datasets. Seventy percent of the invalid responses were randomly allocated to the training dataset, 10% to the validation dataset, and 20% to the held-out test dataset. Evaluated on the held-out test dataset, we found AuDrA’s performance on the invalid-augmented set was quite high (*r* =.94). It is important to note, however, that the enhanced performance was largely driven by AuDrA’s accuracy on the invalid items (MAE =.03). Yet, AuDrA’s accuracy on the invalid test items did not come with a performance tradeoff on the original, valid test items; AuDrA’s correlation with human creativity ratings for the original, valid-item test set (*r* =.80; MAE =.07) was virtually identical to that found in Patterson et al. (2024). Given the enhanced ability to reject invalid responses, we employ the updated version of AuDrA on CAP. Code, items, and updated scores are available on OSF.

## Footnotes

1 CAP was developed using a mixture of Python’s Django package, JavaScript, CSS, and HTML. While the scoring models on CAP were developed by our team and are open-source, CAP’s infrastructure is not; this is due in large part to server security considerations.

2 The reason for walling autoscoring behind study completion is resource-based. Though the CAP server has considerable processing power and storage space, both resources are finite and must be shared among users. Thus, to ensure as many users as possible can have a high-quality experience with CAP, we saw fit to limit autoscoring of studies (which can contain thousands or tens of thousands of responses) to a single occasion after data collection and to enforce autodeletion to maintain space in the CAP database.

## Code availability

All modeling and analysis code for empirical work in this manuscript is available on OSF (https://osf.io/4nkmj/).

## Declarations

### Open practices statement

The data and materials for empirical work in this manuscript (Appendices A–C) are available at https://osf.io/4nkmj/ (modeling/analysis scripts and creativity ratings); this work was not preregistered.
